# Risk factors for femoral stem fracture following total hip arthroplasty: a systematic review and meta analysis

**DOI:** 10.1007/s00402-024-05281-x

**Published:** 2024-04-12

**Authors:** Gareth S. Turnbull, Sam Soete, Muhammad Adeel Akhtar, James Anderson Ballantyne

**Affiliations:** 1 National Treatment Centre-Fife Orthopaedics, Victoria Hospital, Hayfield Road, Kirkcaldy, KY2 5AH UK; 2 The Royal Infirmary of Edinburgh, 51 Little France Cres, Old Dalkeith Rd, Edinburgh, EH16 4SA UK; 3 Department of Trauma and Orthopaedics, The University of Edinburgh, 49 Little France Crescent, Old Dalkeith Road, Edinburgh, EH16 4SA UK

**Keywords:** Stem fracture, Implant failure, Arthroplasty, Modular stem, Implant

## Abstract

**Background:**

Femoral stem fracture following total hip arthroplasty (THA) is an infrequent but nevertheless devastating complication, with an increasing worldwide prevalence as demand for primary THA continues to increase. The aim of this study was to perform a systematic review and meta-analysis of risk factors for femoral stem fracture to help identify at risk patients.

**Methods:**

A systematic search was conducted on EMBASE, MEDLINE and AMED to identify relevant studies. Data regarding study design, source, population, intervention, and outcomes was collated. Data extraction was performed on a custom form generated using Cochrane recommended methodology and analysis of risk factors performed including odds ratios (ORs) with 95% confidence intervals (CIs).

**Results:**

A total of 15 studies reporting a total of 402 stem fractures in 49 723 THAs were identified. The median time from index procedure to stem fracture was 68 months (IQR 42.5–118) whilst mean age at index surgery was 61.8 years (SD 6.9). Male gender (OR = 3.27, 95% CI = 2.59–4.13, p < 0.001), patient weight above 80 kg (OR = 3.55, 95% CI = 2.88–4.37, p < 0.001), age under 63 years (OR = 1.22, 95% CI = 1.01–1.49, p < 0.001), varus stem alignment (OR = 5.77, 95% CI = 3.83–8.7, p < 0.001), use of modular implants (OR = 1.95, 95% CI = 1.56–2.44, p < 0.01) and undergoing revision arthroplasty (OR = 3.33, 95% CI = 2.70–4.1, p < 0.001) were significant risk factors for prosthetic stem fracture. A risk window of 15 years post-surgery was identified.

**Conclusions:**

This review concludes that patient weight, younger age, male sex, varus stem alignment, revision arthroplasty and use of modular stems are significant risk factors for femoral stem fracture. Modifying these risk factors where possible may help reduce incidence of femoral stem fracture in at risk patients.

## Introduction

The reported incidence of femoral stem fracture after total hip arthroplasty (THA) currently ranges from under 0.1 to 3.4%, [1–4] although historically much higher rates have been reported above 10% [5]. The low rate of stem fracture in modern implants has been attributed in part to advances in stem design, metallurgy and cementing techniques [6]. Despite this, rising worldwide demand for THA means the prevalence of stem fractures is expected to increase [7, 8]. Understanding risk factors for stem fracture therefore remains clinically important in order to help minimise risk of this devastating complication to patients.

**Fig. 1:**
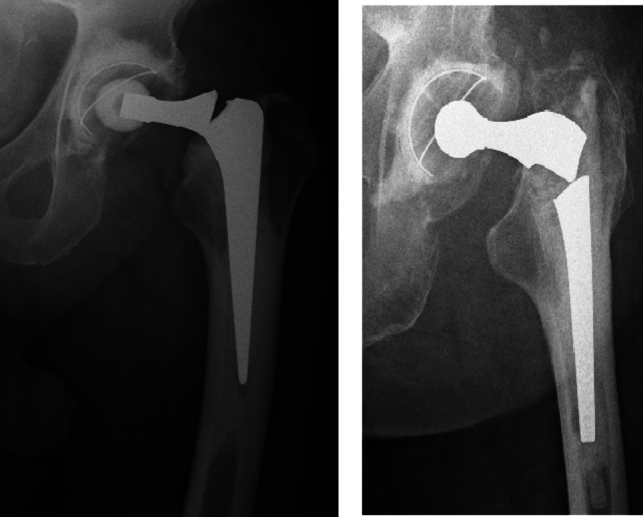
Examples of broken prosthetic stems

Femoral stem fracture is generally thought to occur due to fatigue generated by unfavourable biomechanics. For example, mechanical overload has been recognised to predispose to implant neck fracture [9]. Loss of proximal support with a well-fixed distal stem can also allow repeated cantilever bending and access of body fluid salts to the area of stress. This can promote localized corrosion, fretting and fatigue crack initiation leading to stem failure (Fig. 1) [10]. Previously noted risk factors for stem fracture can be subdivided into three categories with patient, implant and surgical factors all thought to contribute. Patient gender, body mass index (BMI), activity levels and reduced proximal bone stock in context of revision THA have all been noted to increase risk [2, 3, 11]; implant factors including stem design, materials, modularity and reliance on cementless or cemented fixation have also been noted to influence risk [12]; finally, surgical factors including varus malpositioning of the stem, implant undersizing and inadequate cementing technique have also been found to increase risk [1].

Identifying risk factors for stem fracture and modifying them where possible forms part of a wider strategy to help reduce risk of subsequent revision surgery in patients, with revision THA associated with increased costs and poorer outcomes when compared to primary THA [13, 14]. The aim of this study is therefore to perform a systematic review and meta-analysis of risk factors for femoral stem fracture to help identify at risk patients.

## Methods

**Fig. 2:**
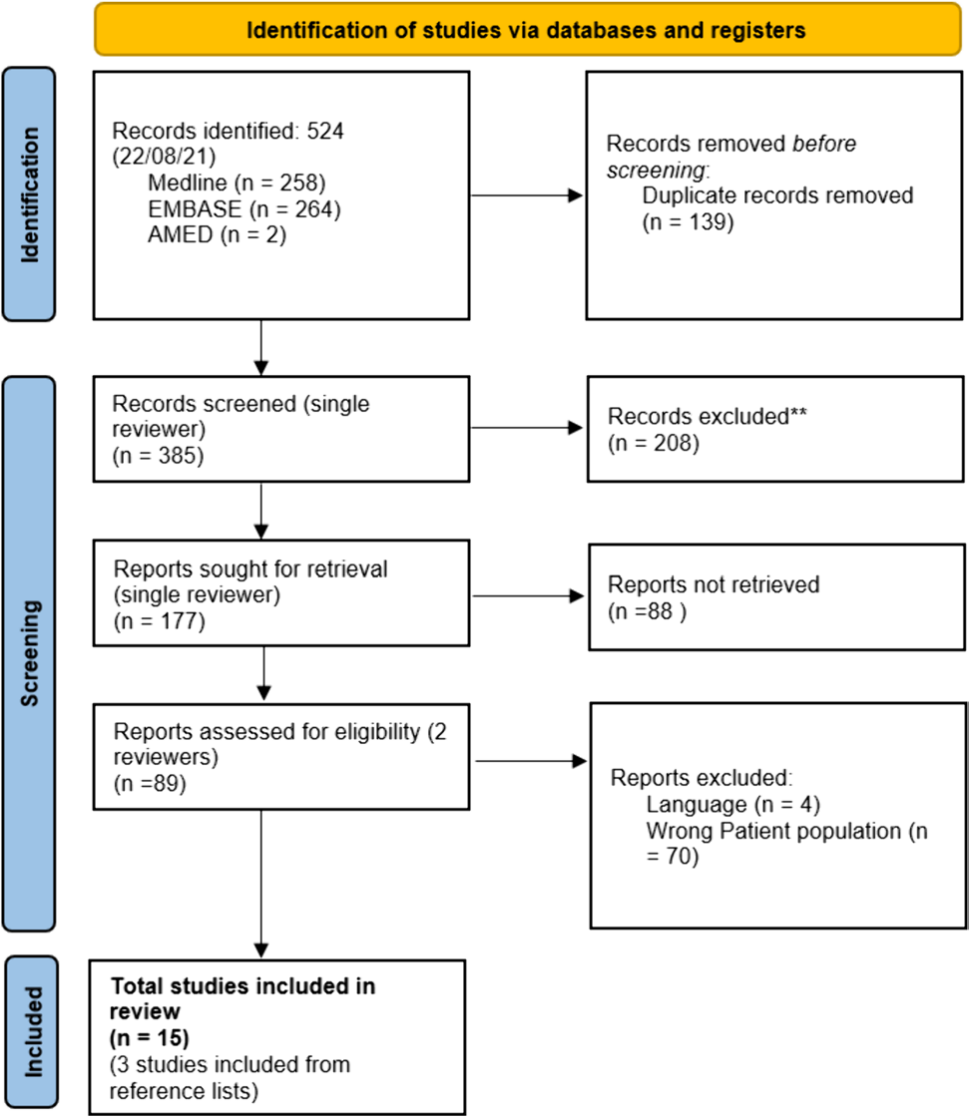
Preferred Reporting Items for Systematic reviews and Meta-Analyses flow diagram showing the study selection process

A systematic literature search was performed for studies that reported femoral stem fracture following THA using selected search terms including arthroplasty, fracture and stem (Fig. 2) The following databases were searched: EMBASE (from 1974), MEDLINE (from 1946) and AMED (1985).

Duplicates were removed and search results reviewed using COVIDENCE software in order to categorize potentially appropriate abstracts. A second full-text screening was performed alongside inclusion and exclusion criteria to identify relevant articles. Reference lists of included papers were also screened to discover any articles that were missed in the initial search.

Studies were excluded if they did not: (1) analyse potential risk factors for prosthetic stem fracture, (2) provide individual participant data on those with stem fractures, (3) analyse the appropriate age group (> 18 years old), or (4) differentiate between stem fracture and dislocation.

### Quality assessment

All included studies were appraised for their quality by two authors using the Critical Appraisal Skills Programme (CASP) checklist specific for cohort studies (Table 1). The assessment tool uses 10 questions to assess study design, validity of results and generalisability to a wider population with the goal of uncovering systematic points of failure [15]. All included studies in this review were observed to be methodologically satisfactory.

**Table 1:** Qualitative assessment of included studies. ( +) indicate positive assessments, empty box represents an inability to answer the question based on presented data, whilst (−) indicates a negative result

CASP cohort study checklist	Did the study address a clearly focused issue?	Was the cohort recruited in an acceptable way?	Were the outcomes accurately measured to minimise bias?	Have they taken account of the confounding factors in the design and/or analysis?	Was the follow up of subjects complete enough?	Was the follow up of subjects long enough?	Are the results precise enough?	Do you believe the results?	Can the results be generalised to a wider population?	Do the results of this study fit with other available evidence?
Amstutz et al. [18]	+	+	+	+	+	+	+	+	+	+
Busch et al. [2]	+	+	+	+	+		+	+	+	−
Krüger et al. [16]	+	+	+	+	+	+	+	+	+	+
Shah et al. [19]	+	+	+	+	+		+	+	+	+
Røkkum et al. [20]	+	+	+	+	+	+	+	+	−	−
Herold et al. [17]	+	+	+	+	+		+	+	+	+
Kishida et al. [21]	+	+	+	+	+		+	+	+	−
Lakstein et al. [1]	+	+	+	+	+	+	+	+	+	+
Matar et al. [22]	+	+	+	+	+		+	+	+	+
Merini et al. [23]	+	+	+	+	+	+	+	+	+	+
Pazzaglia et al. [24]	+	+	+	+	+	+	+	+	+	+
Ritter et al. [25]	+	+	+	+	+		+	+	+	+
Vanbiervliet et al. [26]	+	+	+	+	+	+	+	+	+	+
Wroblewski et al. [11]	+	+	+	+	+		+	+	+	+
Yates et al. [9]	+	+	+	+	+	+	+	+	+	+

### Statistical analysis

This was performed using Statistical Package for Social Sciences version 28.0 (SPSS Inc., Chicago, Illinois). Heterogeneity between studies was tested using pre-operative parameters of age, follow-up duration and sex using the I2 index based on Cochran’s Q with an I2 index greater than 50% deemed heterogenous. Univariate analysis was performed using parametric (Student’s *t*-test: paired and unpaired) and non-parametric (Mann–Whitney U test) tests, as appropriate, to assess continuous variables for significant differences between two groups. Nominal categorical variables were assessed using a chi-squared or Fisher’s exact test. Pearson’s correlation or Spearman’s rank correlation were used to assess the relationship between linear variables as appropriate. Odds rations were calculated to examine the association between stem fracture and different risk factors with corresponding 95% confidence intervals also calculated. The data were standardized to means and SDs, weighted for sample size. A p-value of < 0.05 was considered significant in all analyses.

## Results

There were 385 articles identified in the initial search after duplicates were removed. After primary screening of titles and abstracts, 15 articles meeting the inclusion criteria were identified [2]. The year of publication ranged from 1982 to 2020. Fourteen of the included papers were retrospective studies and one study was prospective in nature. Some studies limited their assessment to an individual prosthesis, whilst others compared the performance of different stem designs (Table 2). Krüger et al. [16], Herold et al. [17] and Yates et al. [9] compared the stem fracture group to a separate control group (Table 2). The number of stem fractures reported in included studies ranged between 3 and 120.

**Table 2:** Summary data of included studies

Year	Author	Total thrs	Stem fractures	Study length	Follow-up time (months)	Description of prosthesis included
1990	Amstutz et al. [18]	716	13	1970–1978	64 (12–180)	Trapezoidal-28 stem. Primary cemented monoblock stainless steel femoral stem
2005	Busch et al. [2]	219	5	Not recorded	Not recorded	Cobalt-chrome diaphyseal fixed revision stems: 151 solution (DePuy) & 68 Echelon (Smith & Nephew)
2020	Krüeger et al. [16]	37,600	110	2010–2017	> 60	Titanium alloy revison stem: MRP-TITAN, Peter Brehm GmbH Titanium alloy, uncemented modular revision femoral stem Demographic data only presented for stem fracture patiets and matched cohort (273 patients in total)
2017	Shah et al. [19]	1177	9	2005–2011	Not recorded	Titanium alloy revison stem: 547 Emperion (Smith & Nephew) & 621 S-ROM (DePuy)
1995	Røkkum et al. [20]	27	3	1983–1985	108–132	Exeter stem, composed of stainless high-nitrogen steel
2021	Herold et al. [17]	1009	32	2002–2017	Not recorded	Revitan stem (Zimmer Biomet GmbH), a titanium alloy modular revision stem
2002	Kishida et al. [21]	204	5	1987–1995	Not recorded	Lubeck, a cobalt–chrome–molybdenum alloy stem used in primary THA
2011	Lakstein et al. [1]	179	6	1999–2009	> 24	Titanium alloy revison stem: ZMR (Zimmer)
2020	Matar et al. [22]	3229	35	2008–2018	Not recorded	15 Polished tapered cemented stems & 10 composite beam & 10 miscellaneous stems
2016	Merini et al. [23]	302	16	2002–2003	10 (1–11)	Hydroxyapetite coated titanium cementless Corail femoral stems with laser neck etching (2nd gen, 2002)
1988	Pazzaglia et al. [24]	365	13	1969–1976	108–192	9 Charnley & 4 Mueller
1986	Ritter et al. [25]	273	14	1974–1980	Not recorded	Stainless steel trapezoidal-28 (Zimmir)
2020	Vanbiervliet et al. [26]	315	7	2010–2017	69	Stainless steel fortress stem
1982	Wroblewski et al. [11]	3983	120	Not recorded	Not recorded	Stainless steel Charnley “flat back”
2008	Yates et al. [9]	125	14	1995–2000	92 with mean 56, 111 with mean 120	Modern, high-nitrogen, stainless steel stems

### Stem fractures

**Fig. 3:**
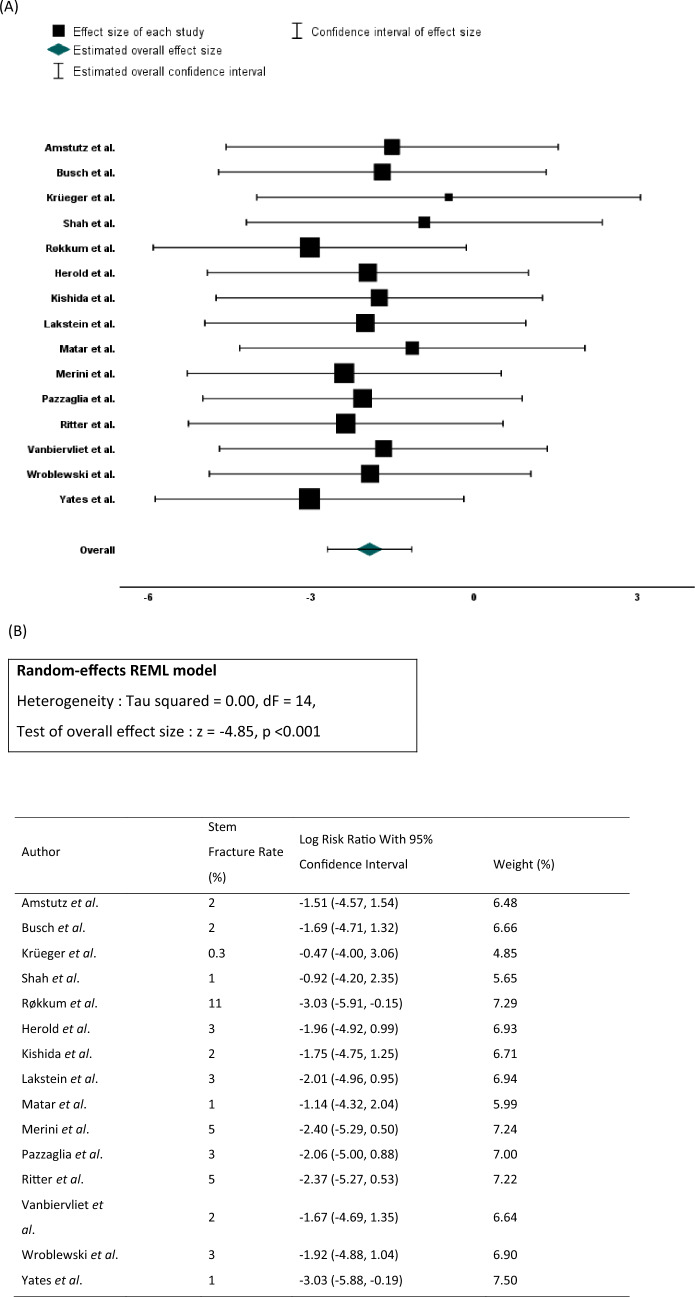
Forest plot (**A**) and corresponding breakdown of random-effects REML model (**B**) examining stem fracture risk of included studies in meta analysis and study weighting

Initial analysis was performed to allow consideration of study weighting and heterogeneity with respect to stem fracture risk. Similar risk profiles were present for stem fracture throughout all included studies (Fig. 3). A total of 402 stem fractures in 49 723 THAs were identified, giving an overall stem fracture rate of 0.8% (range 0.3–11%). The median time from index procedure to stem fracture was 68 months (IQR 42.5–118) whilst mean age at index surgery was 61.8 years (SD 6.9). Whilst operative indication and demographic data was incompletely reported in some studies, osteoarthritis was the most frequent reported indication for index surgery (1538/2185) followed by rheumatoid arthritis (104/2185) and AVN (114/2185). Primary THA was noted in 9539 cases and revision THA in 2857 cases. Male sex was reported in 2110 THAs and female in 2232 THAs. 309/402 stem fractures (77%) across the included studies occurred in male participants.

### Risk factors for stem fracture

**Fig. 4:**
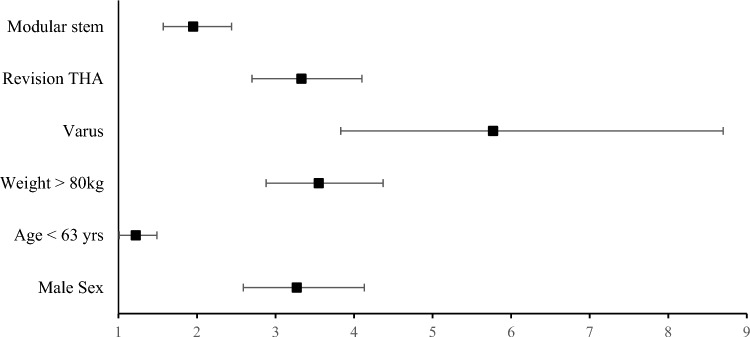
Forest plot of risk factors for stem fracture. Odds ratio and 95% confidence intervals displayed

Several patient factors were found to significantly increase risk for stem fracture on analysing pooled summary data from included studies (Fig. 4). Patients suffering stem fracture were significantly younger (p < 0.05, non-fractured stems age 64.4 ± 6 (SD) years vs fractured stems 63.1 ± 8.3) with those age under 63 years having a significantly increased odds ratio (OR) for suffering stem fracture (OR = 1.22, 95% CI = 1.01–1.49, p < 0.001). Patients suffering stem fracture also had significantly higher average weight (p < 0.05, non-fractured stems 71.1 ± 8 kg vs fractured stems 94.1 ± 16.9) with those above 80 kg having a significantly increased odds ratio (OR = 3.55, 95% CI = 2.88–4.37, p < 0.001). Male gender was also a significant risk factor for stem fracture (OR = 3.27, 95% CI = 2.59–4.13, p < 0.001), with 77% of fractured stems occurring in male patients.

In terms of surgical factors, fractured stems were significantly more likely to be in a varus alignment (OR = 5.77, 95% CI = 3.83–8.7, p < 0.001). Stem fracture was also significantly more likely to occur in the setting of revision THA (OR = 3.55, 95% CI = 2.88–4.37, p < 0.001). Furthermore, use of modular stems also carried increased risk of stem fracture (OR = 1.95, 95% CI = 1.56–2.44, p < 0.01).

## Discussion

The results of our study highlight that several factors predispose to increased risk of femoral stem fracture. Some patient risk factors are clearly non-modifiable, such as male sex and patients requiring THA at a young age. However, there are potential steps that can be taken to reduce risk even in these patients.

The most significant risk factor for fracture on performing meta-analysis appeared to be placing the femoral stem in varus alignment. Previous studies have demonstrated that varus alignment increases the stress placed on the femoral stem [9, 27]. Clinically that has translated in case series to an increased observed rate of stem fracture in those with varus alignment [6, 11, 22, 24, 25]. Our study found that varus alignment acts as a statistically significant risk factor for femoral stem fracture, with 48% of fractured stems having varus alignment. Markolf et al*.* observed a 32.7% increase of bending force in long necks placed in a varus position demonstrating a mechanism for this finding [27]. Contrastingly, Wroblewski et al. noted that stems with valgus alignment fractured significantly sooner than their varus counterparts. However, it was noted that the stems in valgus alignment belonged to heavier patients [11].

Increased patient weight was also found to be a significant risk factor for stem fracture. The role of obesity in increasing patient risk of complications including infection, delayed wound healing, periprosthetic fracture and reoperation has been well described previously [3, 9, 13, 22]. Charnley previously observed a significantly higher stem fracture rate in participants weighing over 88 kg [11]. This is in keeping with our findings of a significant average difference in patient weight of 23 kg between non-fractured and fractured stem groups (71.1 ± 8, 94.1 ± 16.9). Several other case series have also noted obesity as a significant risk factor for prosthetic stem fracture [22, 24, 26, 29].

Patients undergoing revision THA also appeared to be at increased risk of stem fracture. Proximal implant support may be reduced and implant strain increased in revision THA due to bone loss from infection, aseptic loosening, or indeed due to trochanteric osteotomies. If trochanteric osteotomies are indicated in the presence of unsatisfactory proximal bone support, it has therefore been suggested that reinforcement such as in the form of a strut graft is considered [22]. It has also been suggested that the use of small-diameter stems should be avoided in revision THA, especially in patients with other risk-factors for stem fracture such as obesity [22, 24, 29]. Modular implants commonly used in revision THA also had significantly increased risk of stem fracture, in keeping with previous literature. Whilst modular implants allow more greater flexibility in reconstructing the native hip in the setting of bony defects in particular, corrosion at the modular junction has been noted to increased risk of implant fracture and failure [22, 28, 29].

In terms of time to stem fracture, median time from index procedure to fracture was 68 months. Overall, 83% of stem fractures were seen to occur before 10 years, with a very small number occurring beyond 15 years. Wroblewski et al. Previously described an 11-year “at risk” period as the vast majority of fractures within their study occurred within this timeframe [11]. The varying length of follow up performed by the studies in this review makes it difficult to comment on long term stem fracture risk. However, the data available does suggest fracture is a more often a medium rather than short or long term complication to be aware of in at risk patients.

The role patient factors may play in accelerating time to fracture has also been investigated. Wroblewski et al. measured weight gain over time after THA given that weight is not static and can therefore be a dynamic risk factor [11], reporting a linear and significant relationship between weight and time to fracture. However, Krüger et al. observed no significant impact of BMI on the time elapsed post-operatively for stems to break [16]. Our study found a near significant trend towards increased weight leading to quicker stem fracture (r = −0.278, p = 0.08). The influence of other confounding factors was however difficult to account for. For example, patient activity levels are infrequently reported in the literature; this is despite suggestions in some case series that increased activity levels lead to increased stem fracture risk, particularly in younger, heavier male patients [22, 25].

There are limitations to our findings. The heterogeneity of the studies and stems included made it difficult to account for the impact of confounding variables on results. For example, there was a lack of reported data on proposed risk factors for stem fracture including patient activity levels, stem sizing (including stem length, volume and use of higher offset or lateralized components) or indeed occurrence of undersizing, and quality of cement mantle achieved. Limited data was also available on the quality of proximal femoral bone stock in stem fracture patients, which in the context of revision surgery is likely to significantly impact upon the cantilever forces implants are subject to. Due to data limitations, it was also not possible to comment on any impact related to the use of implants being combined from different manufacturers within the same hip construct. Many of the studies included unique measurements of risk factors making it impractical to conduct a meta-analysis on them. Length of follow up was also variable between studies, whilst some stems have been superseded in clinical practice by more modern versions. For example, manufacturer reported fracture rates of the modern Exeter Universal stem are around 0.0006% which is significantly lower than in older versions of the stem [20, 30]. Individual femoral stems are all subject to their own manufacturing processes and individual risk profiles, and it will remain important for the surgeon to remain aware of these during implantation and longer-term follow-up in the future as femoral implants continue to evolve.

In conclusion, this study confirmed several significant risk factors exist for femoral stem fracture. Risk may be minimised by avoiding varus stem alignment, careful use of modular implants in revision THA, and encouraging pre-operative weight loss especially in heavier, young male patients.

## References

[1] Lakstein D (2010). Revision total hip arthroplasty with a porous-coated modular stem: 5 to 10 years follow-up. Clin Orthop Relat Res.

[2] Busch CA (2005). Fractures of distally-fixed femoral stems after revision arthroplasty. J Bone Joint Surg Br.

[3] Heck DA (1995). Prosthetic component failures in hip arthroplasty surgery. J Arthroplasty.

[4] Malchau H (2002). The Swedish Total Hip Replacement Register. J Bone Joint Surg Am.

[5] Martens M (1974). Factors in the mechanical failure of the femoral component in total hip prosthesis. report of six fatigue fractures of the femoral stem and results of experimental loading tests. Acta Orthop Scand.

[6] Köksal A (2020). Femoral stem fractures after primary and revision hip replacements: a single-center experience. Jt Dis Relat Surg.

[7] Kurtz S (2007). Projections of primary and revision hip and knee arthroplasty in the United States from 2005 to 2030. J Bone J Surg Am.

[8] Kurtz SM (2011). International survey of primary and revision total knee replacement. Int Orthop.

[9] Yates PJ (2008). Fractures of modern high nitrogen stainless steel cemented stems: cause, mechanism, and avoidance in 14 cases. J Arthroplasty.

[10] Wheeler K, James L (1971). Fatigue behavior of type 316 stainless steel under simulated body conditions. J Biomed Mater Res.

[11] Wroblewski BM (1979). The mechanism of fracture of the femoral prosthesis in total hip replacement. Int Orthop.

[12] Karachalios T, Komnos G, Koutalos A (2018). Total hip arthroplasty: survival and modes of failure. EFORT Open Rev.

[13] Amin AK (2006). Does obesity influence the clinical outcome at five years following total knee replacement for osteoarthritis?. J Bone Joint Surg Br.

[14] Turnbull GS (2019). Return to activity following revision total hip arthroplasty. Arch Orthop Trauma Surg.

[15] C.A.S.P. (2023) CASP (systematic reviews) checklist

[16] Krueger DR (2020). Mechanical failure of 113 uncemented modular revision femoral components. Bone Joint J.

[17] Herold F, Nötzli H, Eijer H (2021). Short proximal components in modular revision stems carry a higher risk for stem fractures. Hip Int.

[18] Amstutz HC (1990). Stem fracture incidence in trapezoidal-28 stainless steel hip arthroplasty. Clin Orthop Relat Res.

[19] Shah RR (2017). Alarmingly high rate of implant fractures in one modular femoral stem design: a comparison of two implants. J Arthroplasty.

[20] Røkkum M (1995). Stem fracture with the exeter prosthesis. 3 of 27 hips followed for 10 years. Acta Orthop Scand.

[21] Kishida Y (2002). Stem fracture of the cementless spongy metal Lübeck hip prosthesis. J Arthroplasty.

[22] Matar HE (2020). Fractured femoral stems in primary and revision hip arthroplasties revisited: Wrightington experience. J Arthroplasty.

[23] Merini A (2016). Cementless Corail™ femoral stems with laser neck etching: long-term survival, rupture rate and risk factors in 295 stems. Orthop Traumatol Surg Res.

[24] Pazzaglia UE (1988). Failure of the stem in total hip replacement. a study of aetiology and mechanism of failure in 13 cases (1978). Arch Orthop Trauma Surg.

[25] Ritter MA, Campbell ED (1987). Long-term comparison of the Charnley, Muller, and trapezoidal-28 total hip prostheses. A survival analysis. J Arthroplasty.

[26] Vanbiervliet J (2022). High rates of implant fracture of a generic polished tapered femoral stem. Hip Int.

[27] Markolf KL, Amstutz HC (1976). A comparative experimental study of stresses in femoral total hip replacement components: the effects of prosthesis orientation and acrylic fixation. J Biomech.

[28] Fink B (2018). What can the surgeon do to reduce the risk of junction breakage in modular revision stems?. Arthroplast Today.

[29] Fink B, Urbansky K, Schuster P (2014). Mid term results with the curved modular tapered, fluted titanium Revitan stem in revision hip replacement. Bone Joint J.

[30] Ling R (2004). The history and development of the Exeter hip.

